# Endovascular Repair of Traumatic Isthmic Ruptures: Special Concerns

**DOI:** 10.3389/fsurg.2017.00032

**Published:** 2017-06-12

**Authors:** Nikolaos Patelis, Athanasios Katsargyris, Chris Klonaris

**Affiliations:** ^1^First Department of Surgery, Vascular Unit, National and Kapodistrian University of Athens, Laiko General Hospital, Athens, Greece; ^2^Department of Vascular and Endovascular Surgery, Paracelsus Medical University, Nuremberg, Germany

**Keywords:** aortic, isthmic, blunt trauma, rupture, endovascular, TEVAR

## Abstract

Injury of the aortic isthmus is the second most frequent cause of death in cases of blunt traumatic injury. Conventional open repair is related to significant morbidity and mortality. Thoracic endovascular aortic repair (TEVAR) has increasing role in traumatic isthmic rupture, as it avoids the thoracotomy-related morbidity, aortic cross clamping, and cardiopulmonary bypass. Additionally to the technical difficulties of open repair, multi-trauma patients may not tolerate the manipulations necessary to undergo open surgery, due to concomitant injuries. TEVAR is a procedure easier to perform compared to open surgery, despite that a considerable degree of expertise is necessary. Direct comparison of the two methods is difficult, but TEVAR appears to offer better results than open repair in terms of mortality, incidence of spinal cord ischemia, renal insufficiency, and graft infection. TEVAR is related to a—statistically not significant—trend for higher re-intervention rates during the follow-up period. Current guidelines support TEVAR as a first-line repair method for traumatic isthmic rupture. Certain specific considerations related to TEVAR, such as the timing of the procedure, the type and oversizing of the endograft, heparinization during the procedure, the necessity of cerebrospinal fluid drainage, type of anesthesia, and the necessary follow-up strategy remain to be clarified. TEVAR should be considered advantageous compared to open surgery, but future developments in endovascular materials, along with accumulating long-term clinical data, will eventually improve TEVAR results in traumatic aortic isthmic rupture (TAIR) cases. This publication reviews the role, outcomes, and relevant issues linked to TEVAR in the repair of TAIR.

## Introduction

Blunt traumatic thoracic aortic injury of the aortic isthmus is the second most frequent cause of trauma-related mortality (1, 2). Open repair usually consists of a high posterolateral thoracotomy and cardiopulmonary bypass to maintain distal perfusion. This procedure is linked to significant mortality, morbidity, and paraplegia incidence (3–5).

Thoracic endovascular aortic repair (TEVAR) has already been proven to be an accepted option in thoracic aneurysm repair, but it can play a significant a role in other pathologies of the thoracic aorta, such as the traumatic aortic isthmic rupture (TAIR) (6–8). TEVAR demonstrates lower morbidity rates compared to that related to the steps of open repair: thoracotomy, aortic cross clamping, and cardiopulmonary bypass. No commercially available stent-graft exists, which is specifically designed for TEVAR in patients with traumatic thoracic aortic injuries, but off-label use of endografts designed for elective TEVAR cases could potentially be used to treat these gravely injured acute patients. This manuscript reviews the role, outcomes, and relevant issues linked to TEVAR in the repair of TAIR.

## Traumatic Aortic Isthmic Rupture

Isthmus is the segment of the aorta affected in the vast majority of blunt thoracic injury cases, reaching 80% of TAIR patients (9). Other aortic segments, such as the aortic arch, the ascending and the descending aorta are affected significantly less frequently. A number of multiple different forces contribute to the high incidence of TAIR. Posterior movement of the thoracic wall and more specifically of the sternum with simultaneous compression of the aorta onto the spine, sudden hydrostatic pressure increase within the aortic lumen, and massive deceleration altogether lead to shearing and torsion forces on the fixed aortic segments (10). TAIR is related to an approximately 75% mortality rate at the place of injury or during patient transfer to trauma centers, and approximately half of TAIR patients die within the first 24 h after initial medical evaluation (3, 11). The ideal timing for treatment of TAIR patients who reach trauma centers has been constantly changing over the last decades. In the past, these lesions were routinely treated on an emergency basis right after the diagnosis was established, but in recent years, treatment has been shifted toward a more expectant strategy in combination with thorough radiological surveillance and proper pharmaceutical regime, aiming at a strict blood pressure control (12–14).

## Choice of Repair: Endovascular or Open Repair?

Although there is no Level I evidence, TEVAR is gradually gaining ground in treatment of TAIR cases, as the advantages of this procedure in terms of operative complexity when compared to open surgical repair are clear. Open repair usually requires left thoracotomy, single lung ventilation, and aortic cross-clamping with complex cardiorespiratory support. However, in most cases, multi-trauma patients may not tolerate most of the necessary surgical or anesthesiologic periprocedural manipulation, while cervical instability and synchronous presence of multiple fractures could make positioning for left thoracotomy on the surgical table problematic or even impossible (10). Grave concomitant injuries and increased bleeding risk may also render up the use of heparin.

Additionally, multi-trauma patients usually arrive at a hospital with limited or no experience in open surgical treatment of thoracic aortic injuries (3, 15). On the other hand, TEVAR is a more commonly performed procedure and available in most hospitals; therefore it can be more easily applied to TAIR patients. Obviously, TEVAR performed in TAIR patients under emergency circumstances requires considerable endovascular expertise. As the majority of first-line trauma centers do not have appropriately trained surgical teams ready to perform complex open surgery and due to the complexity of the required set-up for extensive surgical thoracic aortic repair, TEVAR can be considered a far more accessible option for treating TAIR patients. Therefore, TEVAR should be considered more applicable compared to open surgery in this group of patients.

Published comparative data between open surgery and TEVAR show a significant advantage of the latter in terms of perioperative morbidity and mortality (5, 13, 16, 17). Perioperative morbidity of open surgery remains high (4, 18) with post thoracotomy pneumonia, paralysis or paresis, and injury to the intrathoracic nerves occurring in 60, 6–30, and 20% of cases, respectively (11, 19–21).

On the other hand, data published in multiple single-center studies reports promising outcomes of TEVAR for TAIR. Feezor et al. reported a 0% 30-day mortality and only one serious endograft-related complication in a series of 22 patients undergoing TEVAR for TAIR (22). In a case series of similar size, Urgnani et al. reported technical success in all 20 cases (100%), no neurological complications and only one (5%) TEVAR-unrelated death (23).

A direct comparison of outcomes between open and endovascular repair for TAIR is difficult. Prospective randomized trials have not been published to date. Systematic reviews and meta-analysis of already published literature is the only source of evidence (24, 25).

The largest systematic review was conducted by Murad et al. (26) under the auspices of the Society for Vascular Surgery (SVS). Data were drawn from 7,768 patients, 77% of which were males. TEVAR was reported to present with a significantly lower mortality rate, compared to that of open surgery (9 vs 19%, respectively, *p* < 0.01). End-stage renal disease (ESRD) and spinal cord ischemia (SCI) incidence was reported to be lower in TEVAR compared to open repair (SCI: 3 vs 9%; ESRD: 5 vs 8%; *p* < 0.01 for all results). Both systemic and graft infections occurred less frequently in patients treated with TEVAR compared to open surgery (Graft: 3 vs 11%; Systemic: 5 vs 13%: *p* < 0.01 for all results). During the follow-up period, TEVAR showed a higher re-intervention trend compared to open surgery, although this trend was not statistically significant (*p* = 0.07). Under the light of the above mentioned findings, the SVS committee published clinical practice guidelines suggesting that TEVAR in patients presenting with traumatic thoracic aortic injuries is linked to better results regarding mortality and morbidity compared to open repair and, therefore, can be considered the first line of treatment (27). This recommendation, however, was based on low-quality evidence (Level C, Grade 2). Up-to-date guidelines published by the European Society of Cardiology (ESC) and the European Society for Vascular Surgery (ESVS) support TEVAR as the primary method for TAIR repair, now based on stronger evidence (Class I, Level B) (28, 29). A recent review of 5 meta-analyses, 2 prospective, and 7 retrospective studies also supported the abovementioned guidelines concluding that TEVAR is the most suitable treatment for TAIR where expertise exists (30).

## Considerations of TEVAR in Isthmic Injuries

TEVAR demonstrates a number of advantages in the treatment of TAIR cases, but some special issues should be addressed and merit further discussion.

### When to Perform TEVAR?

Given the 46% mortality rate noted in non-operatively managed patients with TAIR (26), the SVS committee suggested urgent (<24 h) repair simultaneously or immediately after other injuries have been addressed, but at the latest prior to hospital discharge (27, 31). In some series, TAIR patients underwent delayed TEVAR up to 7–14 days after sustaining the injury and the published data is promising, although the small number of patients in these series might bias the results (32). Expectant strategy with thorough imaging surveillance and proper pharmaceutical treatment might be appropriate for “minimal aortic injury” cases showing limited periadventitial defect or hematoma (32, 33). ESVS suggests that delayed aortic repair for TAIR should be considered only in patients with periaortic hematoma less than 15 mm and when no rupture is present, but better evidence is necessary to support this suggestion (Class IIa, Level C) (29). In contrast with open aortic surgery, TEVAR can be performed in both stable and unstable patients with high technical success rates and good results, making it an attractive modality for unstable patients who urgently need to undergo TEVAR (34).

The timing of aortic repair in patients with TAIR in relation to other injuries should also be addressed. In the majority of TAIR cases, lung contusion, rib fractures, and limb injuries are also present and these can be addressed after the aortic repair (32, 35). Life-threatening injuries such as central neural system damage and/or other uncontrolled bleeding should be addressed probably before any aortic repair. Published data is scarce and more is necessary to answer the question of in what order injuries should be treated in relation to aortic repair.

### TEVAR or Open Repair in Young Patients?

This dilemma has also been addressed by the relevant SVS Committee, which underlined that in acute situations, such as TAIR, patient’s age should not play a significant role in decision-making on the type of repair. Despite the fact that younger patients have been reported to present with a higher risk for late complications, the lower mortality and SCI incidence after TEVAR compared to open repair render these long-term postoperative considerations insignificant (25, 36, 37). On the other hand, younger or fit patients with aortic anatomy unsuitable for TEVAR should consider undergoing open repair (27, 29). To date, it has been accepted that endovascular repair does not have a role in children and teenagers (38). The mismatch between vessel diameter and available stent sizes; the smaller arteries for access and the necessity for surgical exposure of the iliac artery; and finally, the fact that vessels of young individuals will outgrow the placed stents are some of the problems of endovascular repair in children and teenagers. These difficulties may lead vascular surgeons to think twice before proceeding to endovascular repair of isthmic ruptures in such young patients, but successful aortic repair with balloon-expandable stents has already been reported (38).

### Are Currently Available Thoracic Endografts Suitable?

Most of the TAIR patients are of young age, usually younger than 40 years (26). With age, the aorta goes through normal changes such as diameter expansion and decrease of the aortic arch angulation. Available thoracic endografts have been developed to treat aneurysmal disease as they were designed to, and, therefore, they are suitable for larger aortic diameters and less angulated aortic arches. As a result, the “off-label” use of available thoracic endografts in TAIR cases may have anatomic limitations. Poor adaptation of a stent-graft to increased arch angulation could result in bad apposition and sealing, leading to endoleak and migration or collapse of the stent-graft (35, 39). Stent-graft collapse is a life-threatening complication that could lead to acute aortic occlusion and distal organ malperfusion (22). In TAIR cases after TEVAR, stent-grafts are more prone to collapse due to their larger size compared to the smaller aortic diameter of younger patients as mentioned above. Additionally, hypovolemic shock in trauma patients resulting in vasospasm and cyclic diameter variation of 10–20% in synchronization with the heart cycle can result in significant underestimation of the “real” aortic diameter and inaccurate preoperative stent-graft measurements (35, 40). More aggressive oversizing should be applied in gravely hypotensive patients, but it should not exceed 20% (29). All the above pose additional difficulties to optimal stent-graft sizing in TAIR, but endovascular bioengineers have already started to address the need of thoracic endografts that could fit TAIR patients (41).

### Should We Cover the Left Subclavian Artery?

Two of the most controversial issues related to TEVAR is the coverage of the LSA and whether routine or selective LSA revascularization should be preferred. In TAIR cases, the landing zone requirements for TEVAR are different to that of thoracic aneurysmal disease, but the proximity of the isthmic injury to the LSA origin makes coverage necessary in up to 50% of TAIR patients (26, 42, 43). Distal arm ischemia, possible vertebrobasilar pathology, and possible occlusion of thyrocervical collateral arteries to anterior spinal arteries increase the risk of SCI occurrence after LSA coverage. To date, no clear consensus regarding preoperative LSA revascularization has been reached and published data are controversial. Some authors suggest LSA coverage when necessary and expectant strategy, and others suggesting the opposite (32, 35, 44). Suggested indications include patent left internal mammary artery to left anterior descending coronary artery bypass or any anatomic variation that renders a patent left vertebral artery necessary. In any case, decision should be made on an individual basis and take into account the level of expertise in either open or endovascular technique, the patient’s general condition, and the presence of concomitant injuries (27, 45).

### Should TAIR Patients Receive Heparin?

Open TAIR repair with cardiopulmonary bypass requires a large dose of systemic heparin to perform; a disadvantage that TEVAR does not have. Published data partially support performing TEVAR without the use of heparin in TAIR cases with presence of grave concomitant injuries and high risk for bleeding (13, 46). On the other hand, the majority of currently available sheaths are 22–24 F in diameter and occlude the blood flow at the access vessel. Without the use of heparin, this diminished blood flow caused by the sheath could potentially lead to lower limb ischemia, especially when TEVAR procedural time is prolonged by less experienced operators. Routine heparinization in these cases is frequently based on local experience, as available evidence is very limited. Individualizing the decision and balancing the thrombophylic and hemorrhagic potential of each particular patient is currently suggested (27, 32).

### Should We Use Cerebrospinal Fluid (CSF) Drainage?

Spinal cord ischemia occurs rarely (3%) after TEVAR for traumatic aortic rupture, significantly lower than open thoracic aortic repair (26, 47). To our opinion, routine CSF drainage is not justified by a number of characteristics of TEVAR for TAIR, such as the limited length of the covered thoracic aorta and the substantial risk of epidural bleeding in the multi-trauma patient, who frequently presents with synchronous coagulopathy. CSF drainage should be considered only in the presence of SCI symptoms (27). Recognizing signs of SCI in a multi-trauma patient who possibly presents with concomitant TAIR and central and/or peripheral neural system trauma can be challenging, therefore, an objective diagnostic and treatment algorithm should be developed.

### General or Local Anesthesia?

Despite the fact that TEVAR could be performed under local anesthesia in elective cases, in emergency settings with a multi-trauma patient who is often agitated, non-cooperative, and presents with a number of concomitant injuries of various gravity, local anesthesia is less favorable. Published data support that TEVAR for TAIR should always be performed under general anesthesia (27).

### How to Follow-up TAIR Patients after TEVAR?

The ideal strategy for long-term follow-up of TAIR patients after TEVAR is still in evolution. Annually performed CTA control for life is considered the best method for elective TEVAR surveillance, but this strategy might not suit patients who underwent TEVAR for TAIR. Opposite to the nature of degenerative thoracic aortic aneurysms, TAIR is not an evolving aortic disease process, but rather a stable injury as a direct result of trauma. Despite current guidelines suggesting the contrary, annual CTA might not be mandatory if TEVAR in TAIR cases is successfully completed and no complications occurred in the short- and midterm follow-up periods (28). The RESCUE trial results suggest that annual follow-up is mandatory only for a period of 5 years (48). This becomes more important given, the younger age of these patients, and the concerns of cumulative radiation and iodinated contrast exposure (49, 50). Other alternative follow-up strategies have been suggested, such as the combination of plain X-ray and MRA that could be of benefit for the long-term surveillance of these patients (28). Follow-up timing and preferred imaging method after TEVAR in TAIR cases should be individualized, tailored, and adapted to the specific conditions of each particular patient.

### Aortic Anatomy Variations and Anomalies

Anatomic variations and anomalies of the arch and its branches are considered rare, but they are reported to reach as high as 15–34% in published TAIR case series (43). These variations include aberrant right subclavian artery, bovine arch type, Kommerell’s diverticulum, right-sided aortic arch, left vertebral artery, and others. Endovascular specialists should carefully examine the pre-procedural CTA in order to recognize aortic arch anomalies or variations, and consequently avoid complications such as cerebrovascular events, endoleaks, upper extremity ischemia, and SCI. Customized TEVAR materials can address difficult aortic anatomies in elective cases, but in the emergency settings of TAIR, customization is not possible. Therefore, in patients presenting with TAIR and existing arch variations, TEVAR might not be a technically feasible solution, leaving open or hybrid repairs the only way to address the aortic trauma (43).

## Conclusion

Endovascular repair of TAIR should be considered advantageous compared to open repair linked to lower operative mortality, morbidity, and SCI incidence. The quality of evidence though remains relatively low. Besides, current stent-grafts are not designed for use in TAIR and, therefore, they cannot always optimally accommodate the unique anatomy of these young patients. Current key points regarding TEVAR in TAIR can be seen in Table 1. Future developments in endovascular materials along with accumulating long-term clinical data will probably improve the outcomes and enhance the role of TEVAR in TAIR repair.

**Table 1:** Key points.

TEVAR is now accepted as the first line treatment in traumatic aortic isthmic rupture (TAIR) patients TEVAR could be delayed depending on concomitant injuries and patient’s status Open repair might be an acceptable alternative to TEVAR in young TAIR patients Currently available thoracic endografts are not optimized for TAIR patients Left subclavian artery could be covered during TEVAR with special caution to existing collaterals on an individual basis Systemic heparinization for TEVAR in TAIR patients might be avoided if concomitant injury is severe Cerebrospinal fluid drainage should be used upon presence of spinal cord ischemia signs and on an individual basis General anesthesia is often mandatory in agitated and non-cooperative trauma patient No consensus exists on proper follow-up after TEVAR for TAIR Aortic anatomy variations and anomalies might hinder TEVAR in some TAIR patients

## Author Contributions

NP is the corresponding author and has contributed in literature research and manuscript writing. AK has contributed in literature research and manuscript writing. CK has the overall responsibility of the manuscript. All authors have accepted the final (submitted) version of the manuscript.

## Conflict of Interest Statement

The authors declare that the research was conducted in the absence of any commercial or financial relationships that could be construed as a potential conflict of interest.
